# Low-Frequency Model for Radio-Frequency Absorbers

**DOI:** 10.6028/jres.100.018

**Published:** 1995

**Authors:** J. Randa

**Affiliations:** National Institute of Standards and Technology, Boulder, CO 80303

**Keywords:** absorber modelling, absorbing walls, anechoic chambers, EMC modelling, EMI/EMC

## Abstract

A simple model is developed to characterize the behavior of radio-frequency absorbers at low frequency. The absorber is represented by a flat, homogeneous, isotropic slab of lossy material, with effective constitutive parameters. These parameters are determined by a fit to measured data. Excellent fits are obtained in the two applications considered. The model is intended for use in the characterization of absorber-lined chambers at low frequency. It could also be used to predict the low-frequency performance of partially loaded shielded enclosures.

## 1. Introduction

This paper presents a simple model for a wall of (electromagnetic) absorber at low frequency. The motivation for developing the model was the need for a simple representation of absorbing walls which will serve as the basis of a model for characterizing anechoic chambers (ACs) at low frequency. The low-frequency performance of ACs constitutes a serious problem in their use as electromagnetic interference (EMI) test facilities. At high frequency an AC provides a well controlled, isolated, reflectionless environment, but at low frequency the absorber is less efficient, and the walls become (partially) reflective. Depending upon chamber size and absorber size and composition, the deterioration in the performance of a chamber typically occurs between 50 MHz and 500 MHz. Consequently, the frequency range of interest for EMI tests usually extends below the frequency range of good performance for an AC. Nevertheless, ACs are often used for EMI tests at low frequencies because of the lack of good alternatives—particularly for indoor testing of large systems. It is therefore very important that their behavior at low frequency (below about 500 MHz) be characterized and understood. Such a characterization is also needed for planning purposes. Construction of an AC represents a sizable investment; in order to make informed decisions about such a purchase one must know in advance with what accuracy the relevant tests could be performed in an AC of a given size and construction.

The way this problem has typically been approached is to take either measured or calculated values of the reflection coefficient of the absorber and use a geometric optics approximation to calculate the fields resulting from a given source [1–4]. (A finite-difference time-domain analysis of a transmission line chamber was done in [5], for a flat, layered absorber wall.) A modal representation of the fields in the chamber would be more natural than geometric optics for low frequency, but the geometric complexity of the absorbing walls would seem to preclude such an approach. In order to make the mode calculations tractable, we need a simple representation of the behavior of the absorbing walls. That would enable us to solve for the cavity modes and derive an expansion for the fields within the chamber. With the modal expansion we could calculate the fields within the chamber for a given source distribution, compute the difference from the idealized fields assumed in the test of interest, and thereby determine the expected uncertainty in the test as a function of frequency for that chamber.

This paper presents such a model for the characterization of the absorbing walls. The model is *not* intended (and is not suitable) as a tool for absorber design. Models and calculations for absorber design already exist [6–10]. Their approach is to calculate the behavior of absorbing structures from the geometry and the constitutive parameters of the material. We adopt a different approach, which is better suited to our purpose. We do not attempt to predict absorber behavior, but rather to reproduce measured absorber behavior in a model which can be used in subsequent calculations. Instead of starting from measured (or assumed) values of *ϵ*, *μ*, etc., and calculating the properties of the absorber, we start with the measured reflection coefficient of the absorber and adjust the model parameters to agree with the data. In this way we ensure good agreement with the measured (macroscopic) properties of the absorbing wall, and at the same time the model is considerably simpler to use in further calculations than the more sophisticated treatments of [6–10]. We are, however, still reliant on our model to extrapolate from measured reflection coefficient to unmeasured aspects of the absorber’s behavior. Of course, we are also reliant on the quality of the data (and the uncertainty estimates) used in the fits. If the data are wrong, the fitting procedure merely produces a good representation of bad results. This is an important point since measurement of the absorber’s reflection coefficient at low frequency is not a trivial task. The data used in the examples below were taken using a time-domain technique [11, 12], which produces data down to about 20 MHz, but which does not actually measure the true reflection coefficient. For the purpose of demonstrating the application of the model, we assume that the data are results for the reflection coefficient and that any departures from this are included in the uncertainties.

The specific model we use to represent the wall of absorber at low frequency consists of a flat slab of uniform, isotropic, lossy material, backed by a perfect conductor. The approximation of using a *flat* surface is motivated by the fact that structure much smaller than a wavelength cannot be resolved by the incident wave. The relative permittivity, conductivity, and thickness of the slab are left as free parameters, to be determined by a fit to reflection coefficient data. In this paper we take the effective permeability of the absorber to be that of free space, *μ* = *μ*_0_. As will be evident below, the model could also be applied to ferrite tiles, in which case the permeability would be treated as an additional free parameter. The parameters of the model are not the actual physical permittivity, conductivity, and thickness of the foam absorber. Instead they are effective parameters of the flat slab, which include the effect of the size and shape of the absorbing cones. Nevertheless, we expect the effective parameters to be physically “reasonable”; if they are wildly different from the actual physical values, the model is suspect. If a good fit to all relevant data can be obtained with physically reasonable values of the parameters, then we have a simple representation for the absorbing wall at low frequency, which can be used in constructing a model of an AC, a semi-anechoic chamber, or a partially loaded shielded enclosure.

The remainder of this paper will give details of the model and demonstrate that it can successfully represent measured data. The next section derives the reflection coefficients for our model absorbing wall and describes the fitting procedure. Sections 3 and 4 contain applications to reflection coefficient data from two different absorbers. In Sec. 5 we summarize the results and present conclusions.

## 2. Calculation

### 2.1. Reflection Coefficients

**Fig. 1:**
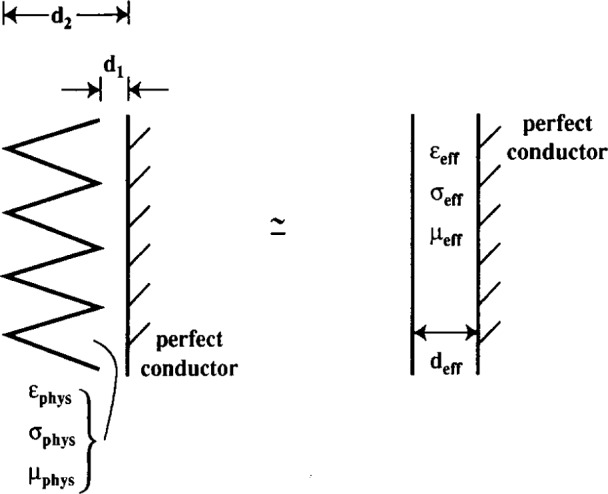
Approximation of replacing absorbing cones with conducting back plane by a lossy slab with a conducting back plane.

The low-frequency approximation underlying the model is pictured in Fig. 1. Since we will be working almost exclusively with the effective parameters, we drop the subscripts on them: *ε*_eff_, *σ*_eff_, *μ*_eff_, *d*_eff_ →*ϵ*, *σ*, *μ*, *d*. Although the applications in this paper will be for nonmagnetic materials, we will not impose *μ* = *μ*_0_ until after we have obtained the general result for the reflection coefficients. Thus our expressions are valid for general (complex) *μ*, and therefore they are applicable to ferrite tiles or composite absorbers, as well as to traditional foam absorbers.

**Fig. 2:**
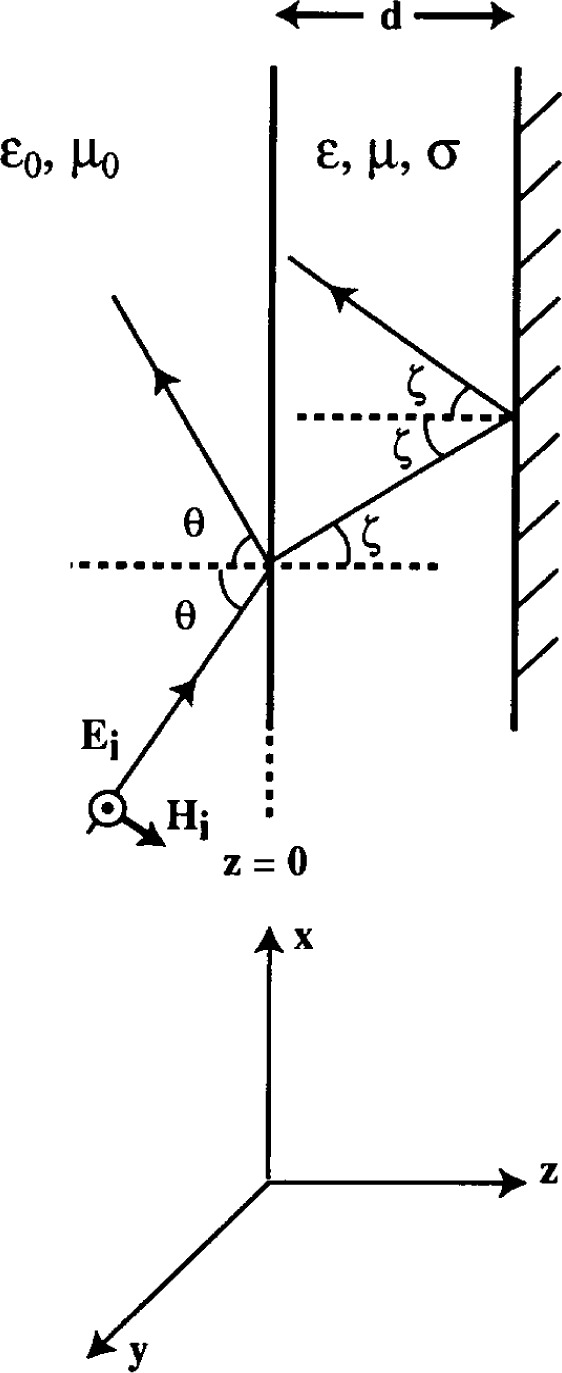
Plane wave incident on lossy slab.

The calculation of the reflection coefficients is a straightforward exercise in boundary matching, with a little complication added by the presence of loss. We assume a monochromatic plane wave (angular frequency *ω*) incident on the absorber at an angle *θ* to the normal. Figure 2 establishes notation and geometric conventions. We have shown the TE case, with ***E*** out of the page. The incident and reflected fields in the air are

$$\begin{array}{l} \;E_{i}=E_{i}\hat{y}e^{-j\beta_{0}\left(x\sin\theta +z\cos\theta \right)},\\ H_{i}=\frac{1}{\eta_{0}}E_{i}\left(-\hat{x}\cos\theta +\hat{z}\sin\theta \right)e^{-j\beta_{0}\left(x\sin\theta +z\cos\theta \right)},\\ E_{r}=E_{r}\hat{y}e^{-j\beta_{0}\left(x\sin\theta -z\cos\theta \right)},\\ H_{r}=\frac{1}{\eta_{0}}E_{r}\left(\hat{x}\cos\theta +\hat{z}\sin\theta \right)e^{-j\beta_{0}\left(x\sin\theta -z\cos\theta \right)},\\ \eta_{0}=\sqrt{\frac{\mu_{0}}{\varepsilon_{0}}},\beta_{0}=\omega \sqrt{\varepsilon_{0}\mu_{0}} \end{array}$$ (1)

where e*^jωt^* time dependence is assumed. Inside the material, the transmitted fields propagating to the right can be written as [13]

$$\begin{array}{l} E_{t}=\hat{y}E_{t}e^{-\alpha z}e^{-j\beta \left(x\sin\zeta +z\cos\zeta \right)},\\ H_{t}=\frac{j}{\mu \omega }E_{t}\left[\hat{x}\left(\alpha +j\beta \cos\zeta \right)-\hat{z}j\beta \sin\zeta \right]\\ \;\times \;e^{-\alpha z}e^{-j}\left(x\sin\zeta +z\cos\zeta \right), \end{array}$$ (2)

where

$$\begin{array}{c} \alpha ={\left[\frac{\omega \mu \sigma }{2}\left(\sqrt{1+{\left(\frac{\omega \varepsilon }{\sigma }\cos^{2}\theta \right)}^{2}-\frac{\omega \varepsilon }{\sigma }\cos^{2}\theta }\right)\right]}^{1/2},\\ \beta =\sqrt{\omega^{2}\varepsilon \mu +\alpha^{2}},\\ \sin\zeta =\frac{\beta_{0}}{\beta }\sin\theta ,\\ \gamma_{z}=\alpha +j\beta \;\cos\zeta . \end{array}$$ (3)

For the waves travelling to the left in the material, we have

$$\begin{array}{c} E_{t^{\prime }}=\hat{y}E_{t^{\prime }}e^{-a\left(d-z\right)}e^{-j\beta \;\left[x\sin\zeta +\left(d-z\right)\;\cos\zeta \right]},\\ H_{t^{\prime }}=\frac{j}{\mu \omega }E_{t^{\prime }}\left[-\hat{x}\left(\alpha +j\beta \cos\zeta \right)-\hat{z}j\beta \sin\zeta \right]\\ \times \;e^{-\alpha \left(d-z\right)}e^{-j\beta \;\left[x\sin\zeta +\;\left(d-z\right)\;\cos\zeta \right]}. \end{array}$$ (4)

Setting the tangential component of ***E*** equal to 0 at the conductor surface (*z* = *d*) and requiring tangential ***E*** and ***H*** to be continuous at the air-material interface yields three equations which can be solved for *E*_r_ in terms of *E*_i_. That yields the reflection coefficient for TE polarization,

$$R_{\text{TE}}\equiv \frac{E_{r}}{E_{i}}=\frac{\mu \omega \cos\theta +j\eta_{0}\gamma_{z}\coth\left(\gamma_{z}d\right)}{\mu \omega \cos\theta -j\eta_{0}\gamma_{z}\coth\left(\gamma_{z}d\right)}$$ (5)

A similar exercise for TM polarization of the incident wave (*H* out of the page) results in

$$R_{\text{TM}}\equiv \frac{H_{r}}{H_{i}}=\frac{\left(\sigma +j\omega \varepsilon \right)\eta_{0}\cos\theta -\gamma_{z}\tanh\left(\gamma_{z}d\right)}{\left(\sigma +j\omega \varepsilon \right)\eta_{0}\cos\theta +\gamma_{z}\tanh\left(\gamma_{z}d\right)}.$$ (6)

Equations (3), (5), and (6) determine the reflection from our absorber model in terms of the parameters *ε*, *μ*, *σ*, and *d*. The next subsection describes the fitting procedure used to determine these parameters.

Before discussing the fitting procedure, one more aspect of the model should be addressed: that is the location of the model slab relative to the actual absorber. Because the thickness of the slab is not the same as that of the absorber (*d*_eff_ ≠*d*_2_ in Fig. 1) there is an ambiguity about whether the front of the slab should coincide with the tips of the cones or whether the conducting back planes of the slab and the cones should coincide—or neither. Resolution of this question would require phase measurements of the reflection coefficients of Eqs. (5) and (6), which are not available at this time. A related question is whether the conducting plane in the model plays the same role as it does in the real case, or whether it also incorporates some of the behavior of the absorber. In particular, for some absorbers the reflections might occur primarily from the planes corresponding to the tips of the cones and to the bases of the cones, with very little energy actually penetrating all the way to the conducting wall. In that case, the conducting wall in our model would perform the function of the transition from cones to continuous backing material. The practical implication of this point is that our model cannot necessarily be blindly extended to the case of absorbing cones without a back plane simply by removing the conducting plane from behind the slab in the model.

### 2.2 Fitting Procedure

The free parameters in Eqs. (3), (5), and (6) are to be determined by fits to experimental data. To perform the fits we used an orthogonal distance regression (ODR) package developed by the NIST Computing and Applied Mathematics Laboratory [14]. As applied in this paper, the ODR procedure is a simple generalization of the ordinary least squares (OLS) fitting procedure. In an OLS fit the residuals are defined as the differences between the fitting function and the measurements at the same frequency, and one minimizes the weighted sum of the residuals squared, which we denote $\chi_{\text{OLS},}^{2}$

$$\begin{array}{c} \chi_{\text{OLS}}^{2}=\underset{i}{\Sigma }{\left[y_{i}-y\left(f_{i};\beta \right)\right]}^{2}w_{i},\\ w_{i}=\frac{1}{\sigma_{i}^{2}}, \end{array}$$ (7)

where *y_i_* are the measurement results, *f_i_* are the measurement frequencies, *y*(*f*, *β*) is the fitting function, *β* represents the free parameters, and the weights *w_i_* are typically taken to be the inverses of the squared standard deviations of the measurements. The ODR procedure defines the residuals as the orthogonal distances between measurement points and fitting curve, thereby allowing for some uncertainty in the measurement frequency. The function to be minimized is

$$\begin{array}{c} \chi_{\text{ODR}}^{2}=\underset{i}{\Sigma }\left(w_{yi}{\left[y_{i}-y\left(f_{i}+\delta_{i};\beta \right)\right]}^{2}+w_{fi}\delta_{i}^{2}\right),\\ w_{yi}=\frac{1}{\sigma_{yi}^{2}},\;w_{fi}=\frac{1}{\sigma_{fi}^{2}}, \end{array}$$ (8)

where *σ_yi_* and *σ_fi_* are the standard deviations in the measurements of *y* and *f*. The data in this paper were all obtained from FFTs of time domain measurements [11, 12], and thus each point represents an average over a significant frequency range (20 MHz) rather than a measurement at one frequency with negligible bandwidth. Consequently an ODR fit is more appropriate for our purposes than OLS. There is not much difference between the two in the examples below, but we will present ODR results unless we indicate otherwise. If the weights *w_i_* are taken to be the inverses of the squared standard deviations (of the means) in Eqs. (7) and (8), then the quantity *χ*^2^ provides a measure of how good the fit is. A correct theory will usually result in *χ*^2^/*ν* ≤ 1, where *ν* is the number of degrees of freedom, defined as the number of measurement points minus the number of fitting parameters. (Those unfamiliar with fitting procedures and significance are referred to references such as [15, 16].)

An important consideration in performing the fits and especially in judging their agreement with the data is the uncertainty (*σ_i_*, *σ_yi_*, *σ_fi_*) ascribed to the measurement results and used in Eqs. (7) and (8). The uncertainty in the frequency is simple enough. We take the distance from midpoint to edge of the frequency bin to be 2*σ_f_*, and thus *σ_fi_* = 5 MHz for all *i*. Each of the reflection coefficient measurements below was performed three times, and we can therefore approximate the random or type-A [17] uncertainty by the standard deviation of the mean, which we call *σ*_A_. In addition, there is a systematic (type-B) uncertainty associated with the method and the instrumentation. These are not very well determined at this time. For this paper, we take them to be about 20 % of the measured reflection coefficient (about 1.6 dB to 2 dB). The full standard deviation in the measurement of the reflection coefficient is then given by [17]

$$\begin{array}{c} \sigma_{i}^{2}=\sigma_{Ai}^{2}+\sigma_{Bi}^{2},\\ \sigma_{Bi}=0.2\left|R_{\text{TE}}\left(f_{i}\right)\right|, \end{array}$$ (9)

at each frequency *f_i_*. This is roughly consistent with the uncertainty estimated for the measurement method in [11, 12]. As will be seen, the additional 20 % uncertainty is not needed to obtain good fits in our examples. We will provide information on *χ*^2^ under both assumptions: *σ*_A_ only and the full *σ_i_* of Eq. (9). In discussing the quality of the fits we will also present figures comparing fit to measurements. In the figures the error bars will correspond only to the statistical standard deviation of the mean of the measurements, *σ*_A_, determined from the spread in the three measurement results at that frequency. As will be seen, the agreement in the figures will also be very good.

Two of our effective parameters, *ε* and *σ*, are frequency dependent. In general, the real and imaginary parts of *μ* are also frequency dependent, but since our applications are nonmagnetic *μ* = *μ*_0_. The effective permittivity and conductivity are parameterized as

$$\begin{array}{l} \frac{\varepsilon \left(f\right)}{\varepsilon_{0}}=1\;{\hat{\varepsilon }}_{100}{\left(\frac{f}{f_{0}}\right)}^{\alpha_{\varepsilon }},\\ \sigma \left(f\right)=\sigma_{100}{\left(\frac{f}{f_{0}}\right)}^{\alpha_{\sigma }}, \end{array}$$ (10)

where *f*_0_ = 100 MHz. Thus $\left(1+{\hat{\varepsilon }}_{100}\right)$ and *σ*_100_ are the values at 100 MHz, and *α_ε_* and *α_σ_* control the frequency dependence. This functional form was chosen for simplicity and convenience; it does not have a physical justification. Indeed, since the frequency dependence of these effective parameters must in some way include the frequency dependent effects of the absorber cones, a physical derivation of the frequency dependence would be quite complicated. In principle, the effective thickness could also have a frequency dependence, but it is not required in our applications, and so we do not include it. We thus have five parameters to vary in our fits: ${\hat{\varepsilon }}_{100}$, *α_ε_*, *σ*_100_, *α_σ_*, and *d*. Given a set of measurement results we vary these five parameters (within physically reasonable values) to minimize *χ*^2^. If the minimum *χ*^2^ corresponds to *χ*^2^/*ν* < 1, then our model agrees with the measured results as well as a full, correct calculation could be expected to agree. Stated another way, the measurements do not distinguish between our model and the full, correct theory. This general procedure is demonstrated (and refined) in the applications in the next two sections.

## 3. Small-Absorber Application

**Fig. 3:**
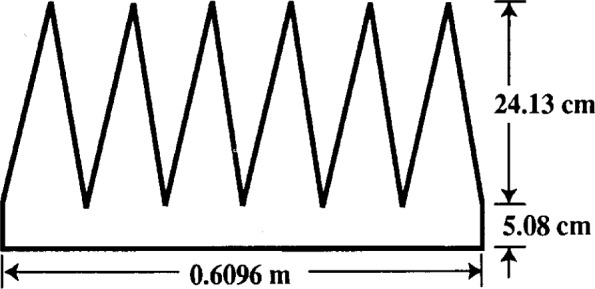
Small absorber used in first application of model.

**Fig. 4:**
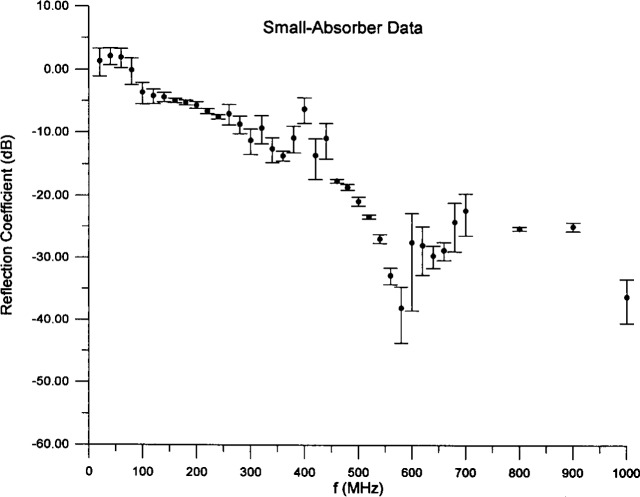
Reflection coefficient data for small absorber.

The first application we consider is to data [18] taken on a small (29.2 cm thick), pyramidal, polyurethane foam absorber, depicted in Fig. 3. The sample used in the measurements was a 2.44 m (8 ft) square. The measurements were made at normal incidence, using the time-domain technique described in [11]. The conversion to the frequency domain introduces a frequency bin size of 20 MHz. Three separate measurements were made, using three different combinations of distances for the transmitting and receiving antennas. From the three measurements we computed an average and standard deviation (using the amplitudes, then converting the results to decibels) at each frequency. The results are shown in Fig. 4. The effective parameters for this absorber are determined by fitting the magnitude of the reflection coefficient, determined from Eq. (5), to the data. All fits and statistics were done on the amplitude |*R*_TE_|, but the results will be presented in decibels. The full *σ_i_* ‘s of Eq. (9) were used in this fit.

There are two aspects of the data of Fig. 4 which invite the injection of some scientific judgment. The first is that |*R*| exceeds 0 dB at low frequency, i.e., more power is reflected than was incident. This unphysical result is an artifact of the measurement method, explained in [11], and we do not want it to influence our fit. Therefore, for points at which |*R*| > 0 dB, we set |*R*| = 0 dB in the fitting routine, and for *σ_i_* we use the greater of the amount by which the measured |*R*| exceeded 1 and *σ_i_* as determined by Eq. (9). The second questionable feature of the data is the behavior around 400 MHz. If we include all those points, the fitting routine attempts to produce a bump around 400 MHz, which does not occur naturally in the model. The result is a poor fit and unrealistic parameter values. In the time-domain method used in the measurements, a spurious structure could have been produced through a conspiracy of inopportune distances, small signals, and the FFT. (This pathology was remedied in later measurements.) We therefore did not include the four data points at 380 MHz–440 MHz in our fits, though we will show them in our comparisons of fits and data. If the structure around 400 MHz proves to be real, our simple model is incapable of reproducing it.

A final consideration in performing the fit is the appropriate frequency range. The model can be expected to fail for frequencies at which the wavelength is comparable to or smaller than characteristic (electrical) lengths of the absorber. In this case the relevant length is twice the thickness of the slab. Due to the taper of the cones, there is a longitudinal dependence of the effective permittivity [3, 6] which is neglected in our model; and when the wavelength in the material becomes comparable to 2*d*, these effects become important. Although we do not know *d* or ε(*f*) before the fit, the dip position tells us the electrical distance in the slab. In the model the first prominent dip is caused by the destructive interference between the waves reflected from the front surface and the back wall. Thus, at the dip frequency the wavelength in the material is about 4*d*, and we can expect the model to break down somewhere in the neighborhood of the first prominent dip, which for this absorber occurs at about 580 MHz.

**Fig. 5:**
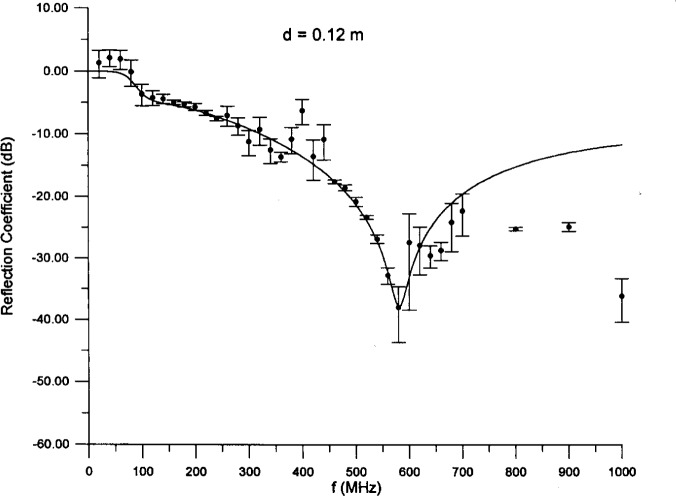
Preferred fit to small-absorber data over 0 MHz–580 MHz frequency range.

For frequency ranges up to about 600 MHz or 700 MHz, the ODR routine has no trouble finding good fits to the measured data of Fig. 4. Indeed, by using different initial values of the parameters it is possible to find many different sets of “optimized” parameters, corresponding to different local minima of the optimization function, Eq. (8). An important point is that we are not necessarily seeking the absolute minimum of the function *χ*^2^. We are more interested in a good fit with realistic parameters than in a slightly better fit with unrealistic values of the parameters. We have found that the most effective way to focus on realistic parameter values is to restrict our search to solutions with thickness *d* between 0.05 m and 0.29 m, since these are the relevant thicknesses of the absorber (see Fig. 3). Good fits were found for 0.05 m ≤ *d* ≤ 0.17 m, but fits for *d* < 0.08 m or *d* > 0.15 m had unpalatably large values of ${\hat{\varepsilon }}_{100}$ and/or *α*_ε_. We therefore narrow the range of good fits to 0.08 m ⩽ *d* ⩽ 0.15 m. For our preferred fit we choose a thickness near the midpoint of the range. The parameter values of the preferred fit are *d* = 0.12 m, *ε*_100_ = 41.30, *α_ε_* = 2.427, *σ*_100_ = 0.009 963 S/m, and *α_σ_* = 0.8008. The curve resulting from using these values in Eqs. (3) and (5) is compared to the data in Fig. 5. All the data are plotted in the figure, not just those used in the fit. The error bars correspond to one standard deviation of the statistical error only, ± *σ*_A_*_i_*. Horizontal error bars are not shown because they are all the same and quite small (65 MHz). It is obvious from the figure that the agreement is excellent over the range of the fit (0 MHz to 580 MHz), and it remains moderately good up to about 700 MHz. To quantify the agreement we note that $\chi_{\text{ODR},}^{2}$ is 2.5 with 20 degrees of freedom, which is embarrassingly good. Even if we include the points near 400 MHz, use *σ* = *σ*_A_*_i_*, and compute $\chi_{\text{OLS},}^{2}$, we obtain $\chi_{\text{OLS}}^{2}/\nu =0.44$ over the 20 MHz to 580 MHz range, which still represents very good agreement.

**Fig. 6:**
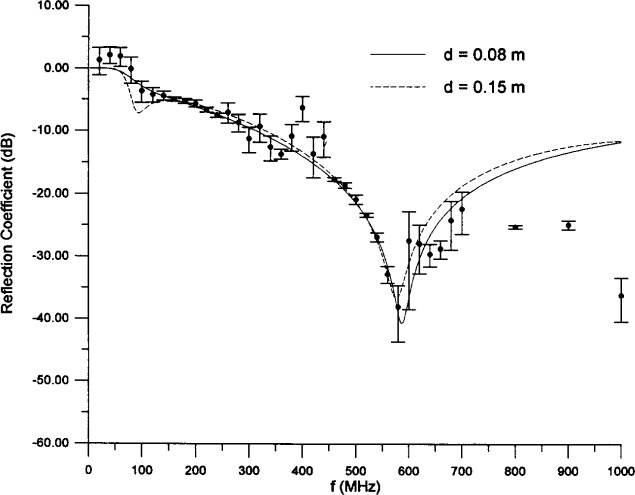
Other acceptable fits to small-absorber data.

We thus have a very good representation of this absorber up to about 600 MHz. It would also be useful to know the uncertainty in the determination of the effective parameters. As noted above, there are good fits with acceptable values of the parameters for 0.08 m ≤ *d* ≤ 0.15 m. The solutions at the ends of this interval are *d* = 0.08 m, ${\hat{\varepsilon }}_{100}$ = 91.29, *α_ε_* = 2.164, *σ*_100_ = 0.012 11 S/m, *α_σ_* = 0.9409, and *d* = 0.15 m, ${\hat{\varepsilon }}_{100}$ = 22.92, *α_ε_* = 2.849, *σ*_100_ = 0.014 29 S/m, *α_σ_* = 0.4439. The curves for these two solutions are compared to the data in Fig. 6, and both agree very well. Therefore, in using this model in a calculation, one would also evaluate the result using (at least) the *d* = 0.08 m and *d* = 0.15 m solutions, in order to gauge the sensitivity of the calculation to the model parameters.

**Fig. 7:**
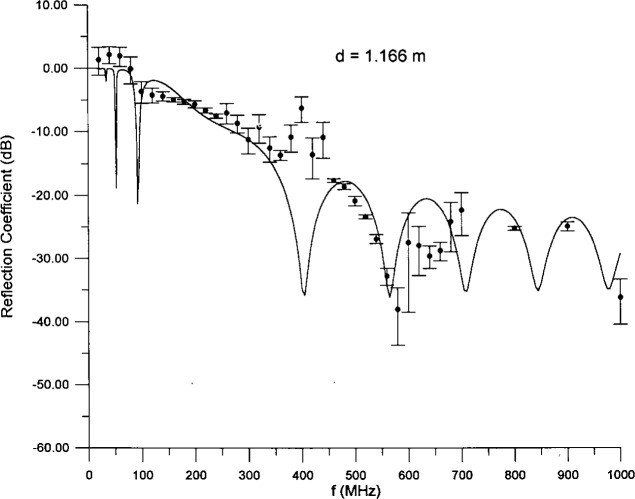
A contrived fit to small-absorber data using unrealistic parameter values.

The exclusion of otherwise good fits because of unacceptable values for one or more parameters warrants comment. Solutions with unrealistic values of the parameters are suspect on the grounds that the parameters may be delicately balanced to produce a fit which agrees with the input data, but produces highly improbable predictions in other places, e.g., at different frequencies or in other measurable quantities. The present model is intended to represent all (macroscopic) facets of the electromagnetic behavior of the absorber, and so we want to minimize the likelihood of nasty surprises. Experience teaches us that avoiding unrealistic parameter values is one way to do so. To illustrate the need for the introduction of human intelligence into the fitting process we present one of the discarded fits, Fig. 7. The parameter values were *d* = 1.166 m, ${\hat{\varepsilon }}_{100}$ = 18.07, *α_ε_* =2.930, *σ*_100_ = .004411 S/m, *α_σ_* =0.3016. The fitted value for *d* is about 4 times the physical thickness of the absorber, but the fit is quite good (ignoring the suspect 380 MHz to 440 MHz points), $\chi_{\text{ODR}}^{2}/\nu =0.75$. However, it is clear that the fit would not survive measurements at additional low frequencies or around 400 MHz.

## 4. Application to Mid-Size Absorber

**Fig. 8:**
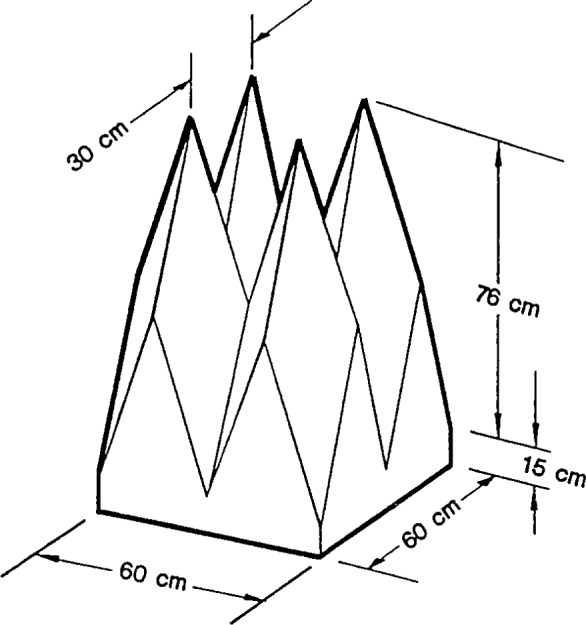
Mid-size absorber used in second application.

The second example is absorber 3 of [11]. It is a fire retardant absorber, 0.91 m tall, with twisted pyramids, Fig. 8. The test sample was a 3 m square. Additional measurements were made on this absorber after publication of [11], and we now have three different measurements at each frequency. We also present more frequency points than previously published. As in the first example, the three measurements are used to compute an average and standard deviation. In this case there are no obvious anomalies requiring special treatment. Fits were performed using both the full *σ_i_* of Eq. (9) and using just the statistical standard deviation *σ*_A_*_i_* computed from the three measurements. We will present the fits obtained using just the *σ*_A_*_i_*. Drawing on the experience of the last example, we chose the frequency range to extend to 300 MHz, just beyond the first prominent dip.

**Fig. 9:**
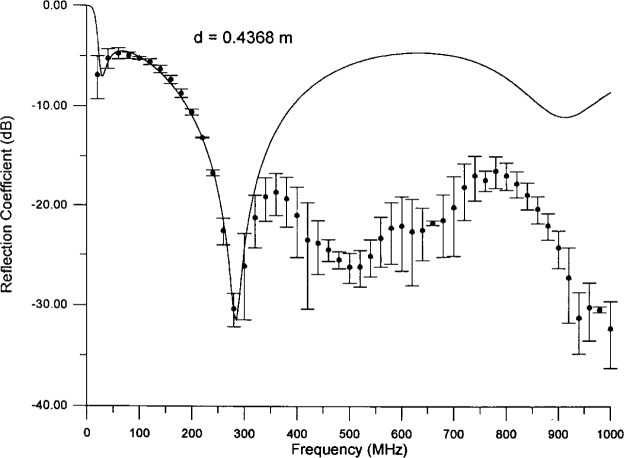
Preferred fit, over 0 MHz–300 MHz range, to data for mid-size absorber.

The absorber dimensions suggest that the effective thickness *d* should be between 0.15 m and 0.91 m. We scanned this entire region and were able to find good fits for 0.37 m ≤ *d* ≤ 0.55 m. Fits with *d* less than about 0.40 m have a very deep, sharp dip between 20 MHz and 40 MHz, and consequently we discarded them. Figure 9 compares the fitted curve to the data for the best fit, *d* = 0.4368 m, ${\hat{\varepsilon }}_{100}$ = 69.08, *α_ε_* = 2.049, *σ*_100_ = 0.018 61 S/m, *α_σ_* = −0.4267. The agreement is very good, as is reflected by the fact that $\chi_{\text{ODR}}^{2}/\nu =0.27$, using just *σ*_A_. If we wish to avoid negative values of *α_σ_*, we can fix *α_σ_* = 0. Then the best fit is for *d* = 0.4568 m, ${\hat{\varepsilon }}_{100}$ = 63.31, *α_ε_* = 2.062, *σ*_100_ = 0.01267 S/m, for which $\chi_{\text{ODR}}^{2}/\nu =0.89$.

There are also data for the angular dependence of the reflection coefficient for this absorber [12]. (Again, what is measured is an approximation to the true reflection coefficient.) Because the time windowing was different for the bistatic angular measurements (front surface reflection as opposed to full reflection), we did not include these data in the fit. We can compare calculated results to angular data using the fitted values for the effective parameters, but there is a minor difficulty in doing so. The *θ* = 0 bistatic data do not agree exactly with the monostatic data. Since the model was constrained to agree with the monostatic data, its overall normalization will not be correct for the bistatic (angular) data. This is seen in Figs. 10a and 10b, where the dashed curves are the model calculations using the parameters noted above (*d* = 0.4368 m, etc.). The vertical error bars on the data correspond to ±2 dB [12], whereas the horizontal error bars reflect the size of the angular bins. For purposes of comparing the shape of the angular calculations to the data, we have also plotted a solid curve which is the model calculation normalized to agree with the angular data at *θ* = 0, as it would if bistatic and monostatic measurements agreed exactly. The agreement for the two representative frequencies shown is quite satisfying and offers additional evidence for the basic validity of the model.

## 5. Summary and Conclusions

We have presented a simple model for the low-frequency behavior of absorbing materials backed by a conducting wall. The model does not enable one to calculate absorber properties from geometrical and material properties, and therefore it is not an absorber design tool. It is intended instead as a way to incorporate measured properties of absorbers in calculations of the performance of chambers lined entirely or partially with those absorbers. (It could also provide a simple parameterization of calculated properties.) The model was applied to two absorbers of different sizes and shapes and was able to replicate the behavior of their reflection coefficients up to a frequency slightly above the first prominent minimum in each case. The two examples considered were polyurethane foam, but the model should also be applicable to ferrite or composite absorbers. There are two principal advantages of the model. One is its simplicity, which should permit its use as the basis of relatively realistic and detailed calculations of field structure within absorber-lined chambers. The other advantage is that (by construction) it accurately reproduces the measured reflection properties of walls of absorbing material. Of course, this is an advantage only if one has good measurements of the reflection properties.

There are several obvious possibilities for further work. One direction is additional model development and validation. Topics which could be addressed comprise additional fits to traditional pyramidal absorbers, use of phase information to determine the appropriate position of the model absorber relative to the real absorber, inclusion of bistatic data in the fits, expansion of the frequency range of the fits (e.g., by letting *d* vary with frequency) and application to ferrites. An interesting and economical way to address some of these questions would be to fit the model to data generated by a more sophisticated and predictive model, such as [6–10]. The other obvious direction for future work is to use this model for its intended purpose—characterization of anechoic chambers, semi-anechoic chambers, and partially loaded screened rooms. Now that we have a relatively simple planar model for a wall of absorber, the electromagnetic problem of calculating a modal expansion for the field distribution in the chamber for a given source distribution should be considerably less daunting.

## Figures and Tables

**Fig. 10a:**
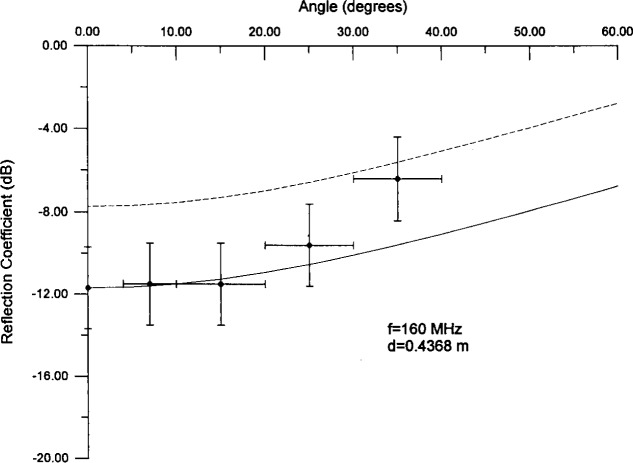
Predicted curves for angular dependence of reflection coefficient at 160 MHz. Solid curve has been normalized to take into account the discrepancy between monostatic and bistatic data.

**Fig. 10b:**
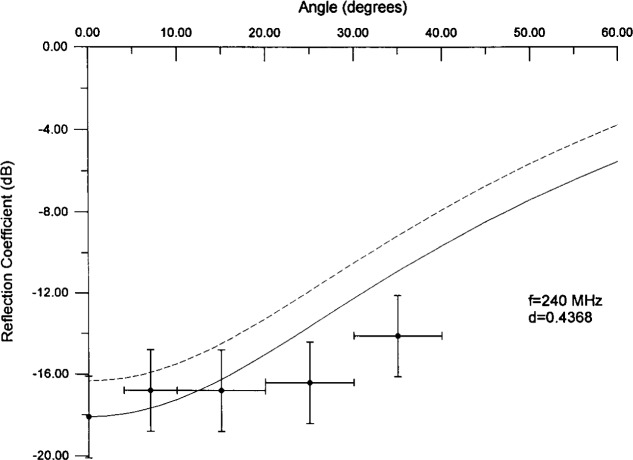
Predicted curves for angular dependence of reflection coefficient at 240 MHz. Solid curve has been normalized to take into account the discrepancy between monostatic and bistatic data.
